# Recurrence and Postoperative Death in Patients with Colorectal Cancer: A New Perspective via Semi-competing Risk Framework

**DOI:** 10.5152/tjg.2023.22540

**Published:** 2023-07-01

**Authors:** Malihe Safari, Leila Mahmoudi, Emma K. Baker, Ghodratollah Roshanaei, Ramazan Fallah, Ali Shahnavaz, Mohammad Asghari-Jafarabadi

**Affiliations:** 1 Department of Biostatistics, Arak University of Medical Sciences Faculty of Medicine, Arak, Iran; 2 Department of Statistics and Epidemiology, Zanjan University of Medical Sciences Faculty of Medicine, Zanjan, Iran; 3 Cabrini Research, Cabrini Health, Melbourne, Australia; 4 Department of Biostatistics, Modeling of Non-communicable Diseases Research Center, Hamadan University of Medical Sciences Faculty of Public Health, Hamadan, Iran; 5 Department of Mathematics and Statistics, Islamic Azad University Zanjan Branch, Zanjan, Iran; 6 Road Traffic Injury Research Center, Tabriz University of Medical Sciences, Tabriz, Iran; 7 Department of Nursing and Health Sciences, Monash University Faculty of Public Health and Preventative Medicine, Melbourne, Australia

**Keywords:** Recurrence, survival, colorectal neoplasms, risk, statistical model

## Abstract

**Background/Aims:**

Cancer studies suffer from an overestimation of prediction of survival when both recurrence and death are of ­interest. This longitudinal study aimed to mitigate this problem utilizing a semi-competing risk approach evaluating the factors ­affecting recurrence and postoperative death in patients with colorectal cancer.

**Materials and Methods:**

This longitudinal prospective study was conducted in 284 patients with resected colorectal cancer who were referred to the Imam Khomeini Clinic in Hamadan, Iran, during 2001-2017. Primary outcomes were postoperative outcomes and patient survival, including time to recurrence (of colorectal cancer), time to death, and time to death after recurrence. All patients who were alive at the end of the study were censored for death and who did not experience recurrence of colorectal cancer were also censored for recurrent colorectal cancer. The relationship between underlying demographics and clinical factors and the outcomes was assessed using a semi-competing risk approach.

**Results:**

The results of the multivariable analysis showed that having metastasis to other sites (hazard ratio = 36.03; 95% CI = 19.48-66.64) and higher pathological node (pN) stage (hazard ratio = 2.46; 95% CI = 1.32-4.56) were associated with a raised hazard of recurrence. The fewer chemotherapies (hazard ratio = 0.39; 95% CI = 0.17-0.88) and higher pN stages (hazard ratio = 4.32; 95% CI = 1.27-14.75) showed significantly higher hazards of death without recurrence. Having metastasis to other sites (hazard ratio = 2.67; 95% CI = 1.24-5.74) and higher pN stages (hazard ratio = 1.91; 95% CI = 1.02-3.61) were linked with the higher hazard of death after recurrence.

**Conclusion:**

Considering findings on death/recurrence-specific predictors obtained in this study to manage the outcomes in patients with colorectal cancer, tailored strategies for preventive and interventional plans should be deliberated.

**Main Points**

- Most cancer studies suffer from an overestimation of prediction of survival when both recurrence and death are of interest.
- Semi-competing risk approach can mitigate the overestimation problem, providing with the probability of recurrence, the probability of death without recurrence, and the probability of death after recurrence.
- Metastasis to other sites and higher pN stage were associated with a raised hazard of recurrence.
- The fewer chemotherapies and higher pN stages showed significantly higher hazards of death without recurrence.
- Having metastasis to other sites and higher pN stages were linked with the higher hazard of death after recurrence.

## INTRODUCTION

Colorectal cancer (CRC) is the most common gastrointestinal malignancy^1^ and is expected to increase by 60% to 2.2 million new cases and 1.1 million deaths globally by 2030.^2^ Colorectal cancer is the second leading cause of cancer-related death globally, being the second and third leading cause of cancer-related death in men and women, respectively.^3^ The incidence rate of CRC is increasing worldwide, especially in developing countries.^4,5^ Thus, there is an urgent need to identify the factors that underlie poor outcomes and drive the recurrence in order to guide appropriate treatment and survival strategies.

Although surgery is the primary treatment, the recurrence rate in the first 5 years after surgery is 12.8% for local recurrence and 25.6% for distant metastasis,^6^ with about 60%-80% of recurrences within 2 years after resection.^7^ Poor survival is associated with a short recurrence interval^8^ and may be improved by curative surgical resection if diagnosed early.^9^ The main goal of follow-up programs after CRC treatment is to increase survival, and early diagnosis increases the likelihood of patients’ survival.^10^

To assess the predictors of recurrence and survival in patients with CRC, Lin et al^11^ investigated the clinical features and risk factors of patients with CRC oligo-metastases of the liver by curative resection. Node-positive primary tumor (pN stage) and metastasis were found to significantly affect recurrence. Farhat et al^7^ investigated the predictors of recurrence in patients with rectal cancer after curative resection, identifying distal resection margin, extracapsular invasion, tumor stenosis, and degree of parietal invasion as prognostic factors. Gunawardene et al^12^ found that the stage of the disease was related to recurrence after surgery. In the study by Duineveld et al.^13^ the type of visit and the tumor location significantly affected the type of recurrence. Saso et al^14^ showed that tumor-related characteristics, including preoperative serum carcinoembryonic antigen level, preoperative obstruction, tumor invasion, lymphatic invasion, and venous invasion, were significantly associated with disease-free survival. In a 5-year cohort study by Zare-Bandamiri et al^15^ the effects of age, tumor location, lymphovascular invasion, and tumor stage on patient recurrence were significant. Another study found that in patients with CRC, high carcinoembryonic antigen level and lymphovascular invasion factors and in patients with rectal cancer, factors including liver metastasis and venous invasion were identified as risk factors for recurrence.^16^ Yazilitas et al^17^ investigated the relationship of pathological and clinical features with time to recurrence in patients with early-stage CRC, identifying that grade I and superficial tumors (T1-T2) are predictors of late recurrence. Tsikitis et al^18^ examined predictors of relapse-free survival in patients with CRC in stages II and III and reported that only T stage was related to CRC recurrence. Therefore, various factors may affect the recurrence and the time interval between recurrence and death, and there is no appropriate agreement for the predictors.

Competing risks occur frequently in the analysis of survival data. Semi-competing risk framework refers to the general setting where the main scientific concern lies in estimation and inference regarding a nonterminal event (e.g., recurrence), the occurrence of which is dependent on a terminal event (e.g., death).^19^ However, due to a strong relationship between the 2 event times, the typical survival modeling for the nonterminal event will cause an overestimation of outcome probabilities.^20^ A solution has been suggested in the competing risk framework,^21,22^ which does not consider the dependence of the 2 events. However, the semi-competing risk analysis framework appropriately treats the dependence between nonterminal and terminal events as a part of the model specification.^23^ Cancer studies suffer from an overestimation of the effects of the predictors on survival when both recurrence and death are of interest. This longitudinal study aimed to mitigate this problem utilizing a semi-competing risk approach evaluating the factors affecting recurrence and postoperative death in patients with CRC. Based on an extensive search in the literature, we did not find any study that uses this framework to assess the outcome of patients with recurrent CRC.

## MATERIALS AND METHODS

### Study Design and Setting

This longitudinal prospective study included a cohort of 284 patients after CRC curative resection who were admitted to Imam Khomeini Clinic in Hamadan, Iran, during 2001-2015. The patients were followed until August 2017, and the data were analyzed accordingly. Patients who were diagnosed with colorectal cancer and had undergone surgery for colorectal cancer were included in this study. Patients undergoing transanal surgery were excluded. Data on patient demographics, treatment, mortality and morbidity, and survival were collected.

### Predictors

All demographic and clinical/pathological information were extracted from patients’ medical records and administrative resources. These included demographic variables such as age at diagnosis (years), gender (female: 1; male: 2), body mass index (kg/m^2^), and clinical/pathological variables such as metastasis to other sites (no: 0; yes: 1), cancer site (colon: 1; rectum: 2), surgery (no: 0; yes: 1), radiotherapy (no: 0; yes: 1), chemotherapy (no: 0; yes: 1), number of chemotherapy (0: no; 1: <6; 2: 6+), morphology (0: no adeno; 1: adeno), grade of differentiation (1: well; 2: moderate; 3: poor), tumor size (1: <4; 2: ≥4 < 7; 3: ≥7), cancer stage (1: B; 2: C; 3: D), pathological node (pT) stage (1: T2; 2: T3; 3: T4; 4: TX [Main tumor cannot be assessed due to lack of information]), and pN stage (1: N2; 2: N3; 3: N4; 4: NX [Regional lymph nodes cannot be assessed due to lack of information]). The cancer stage was adopted according to the American Joint Committee on Cancer staging handbook.^24^

## Main Outcome Variables

Primary outcomes were postoperative outcomes and patient survival, including time to recurrence (of CRC), time to death, and time to death after recurrence. All patients who were alive at the end of the study were censored for death and who did not experience recurrence of CRC were also censored for recurrent CRC. Patients’ recurrence status was determined from the patient records for those patients who experienced CRC recurrence, which was computed from the date of surgery to local/distant recurrence in months. The CRC recurrence was defined as coming back of the disease after treatment, which was determined by the patients’ doctor. When cancer shows up in the same/different organs (like the liver or lungs) similar to the first time it happened, it is called a local/distant recurrence. Besides, the time to death was computed from the date of surgery to the patients’ death. The follow-up was done by the main researcher via a telephone call to confirm the patients’ vital status (death, recurrence, or alive) in August 2017. After this time, the data were extracted from the hospital database and considered for the process of analyses.

### Ethics Committee Approval

The institutional review board of Tabriz University of Medical Sciences approved the protocol of the study (ethics code: IR.TBZMED.REC.1400.457). The participants’ privacy was preserved. All the processes were in accordance with international agreements (World Medical Association, Declaration of Helsinki, Ethical Principles for Medical Research Involving Human Subjects).

### Informed Consent

All participants, or their legal guardian, provided informed written consent on registration in the database. Also, all methods were carried out in accordance with relevant guidelines and regulations.

### Statistical Analysis

**Figure 1.**
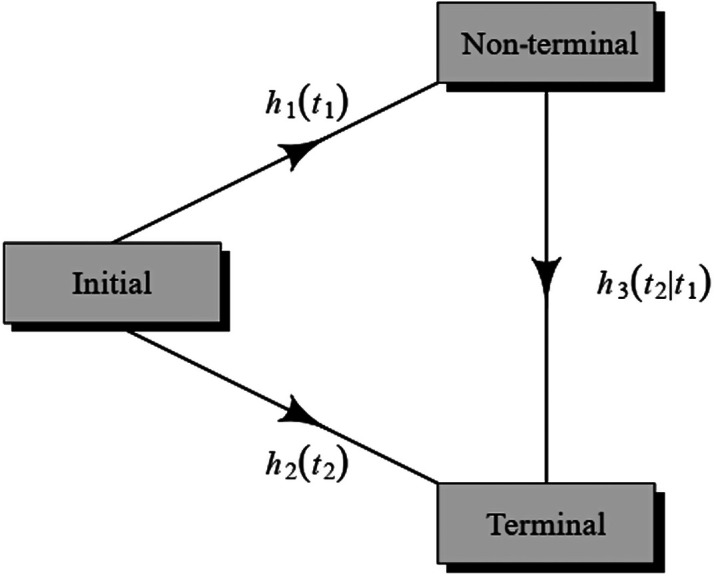
Graphical representation of semi-competing risks.

Data are summarized and reported as mean (SD) and median (minimum–maximum) for the normal and non-normal numeric variables, respectively, and as frequency (percent) for categorical variables. The recurrence/death rates were computed per 1000 persons. Log-rank tests were carried out to compare the survival rates across groups. Primary outcomes were considered as recurrence (of CRC) (called nonterminal event), time to death (called terminal event) without recurrence, and time to death after recurrence. To model the specific risk factors of recurrence and death, semi-competing risk analysis was utilized under the illness-death multi-state model. We utilized proportional hazards models characterized by 3 hazard functions, illustrated in Figure 1.

The following specification for hazard functions was considered:

1. a cause-specific hazard for the nonterminal event, 
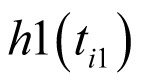
 :



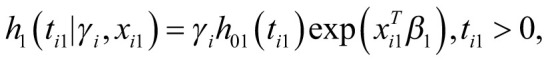



2. a cause-specific hazard for the terminal event, 
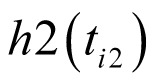
 :



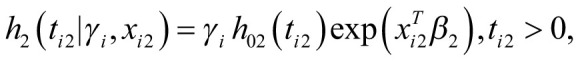



3. and a hazard for terminal event conditional on the time for the nonterminal event, 
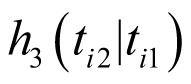
 :



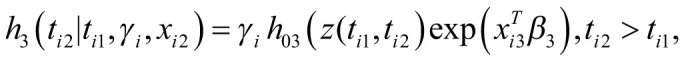



where *h*_0_ *_g_* is an unspecified baseline hazard function and *β_g_* is a vector of log-hazard ratio (HR) regression parameters associated with the covariates 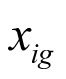 , and 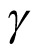 *_i_* is a study subject-specific shared frailty following a Gamma(*θ* ^−1^, *θ* ^−1^) distribution, parameterized so that E[ 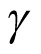 *_i_*] = 1 and Var[ 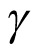 *_i_*] = *θ*.

In our study setting, equations 1-3 are the probability of recurrence of CRC, the probability of death from CRC without any recurrence, and the probability of death from CRC after any recurrence, respectively. To model the risk factors with these outcomes, in univariable and multivariable terms, HRs of the outcomes were estimated using the R 4.2 software utilizing the SemiCompRisks package.^23^ The significance level was set at .05.

## RESULTS

### Patient Population Profile

Of a total of 284 patients (52.8% male) with resected CRC, 131 (46.1%) had a recurrence, of which 105 (80.2%) died by the end of the study. A total of 121 (42.6%) patients died by the end of the study, regardless of recurrence. The mean age at diagnosis of the patients was 55.6 (SD 13.1, range: 21-84) years. Moreover, the 1-, 3-, 5-, and 10-year survival probabilities were 86.9%, 62.1%, 50.4%, and 42.3%, respectively, for terminal events, and were 67.4%, 51.9%, 45.3%, and 40.3%, respectively, for nonterminal events. After CRC recurrence, 1-, 3-, 5-, and 10-year survival probabilities were 67.4%, 51.9%, 45.3%, and 40.3%, respectively (see Table 1 for more information).

### Outcome Rates

For both recurrence and death, significantly higher outcome rates were observed among higher age categories, with substantially higher rates in the age group above 70 years. Patients with metastases to other sites also had much higher rates of both outcomes. In addition, patients who had received less than 6 types of chemotherapy were associated with higher outcome rates for both recurrence and death; however, the rates decreased for patients who had received more than 6 chemotherapies. Nonterminal and terminal event rates raised significantly as the disease stage, pT stage, and pN stage levels, increased (all *P* < .05) (see Table 2 for more information).

### Univariable Semi-competing Risk Model

The study results indicated significant and positive associations between upper age categories with a higher hazard of recurrence (HR = 2.06) and death without recurrence (HR = 3.62). Metastasis to other sites was linked with a raised risk of recurrence (HR = 36.26) and death after recurrence (HR = 2.56). The higher number of chemotherapies significantly decreased both death without recurrence (HR = 0.39) and the hazard of death after recurrence (HR = 0.30). Also, a poor level of differentiation was connected with the outcome of death after recurrence (HR = 3.64). Patients with higher disease stages had significantly higher hazards for all 3 outcomes occurring (all HR >4). Similar results were observed for pT stage and pN stage (Table 3).

### Multivariable Semi-competing Risk Model

Metastasis to other sites was associated with a raised hazard of recurrence (HR = 36.03) and the hazard of death after recurrence (HR = 2.67). The higher number of chemotherapies significantly decreased the hazard of death without recurrence (HR = 0.39). Also, patients with a higher pN stage showed significantly higher hazards of all 3 outcomes occurring (all HR > 1.9) (Table 4).

**Figure 2.**
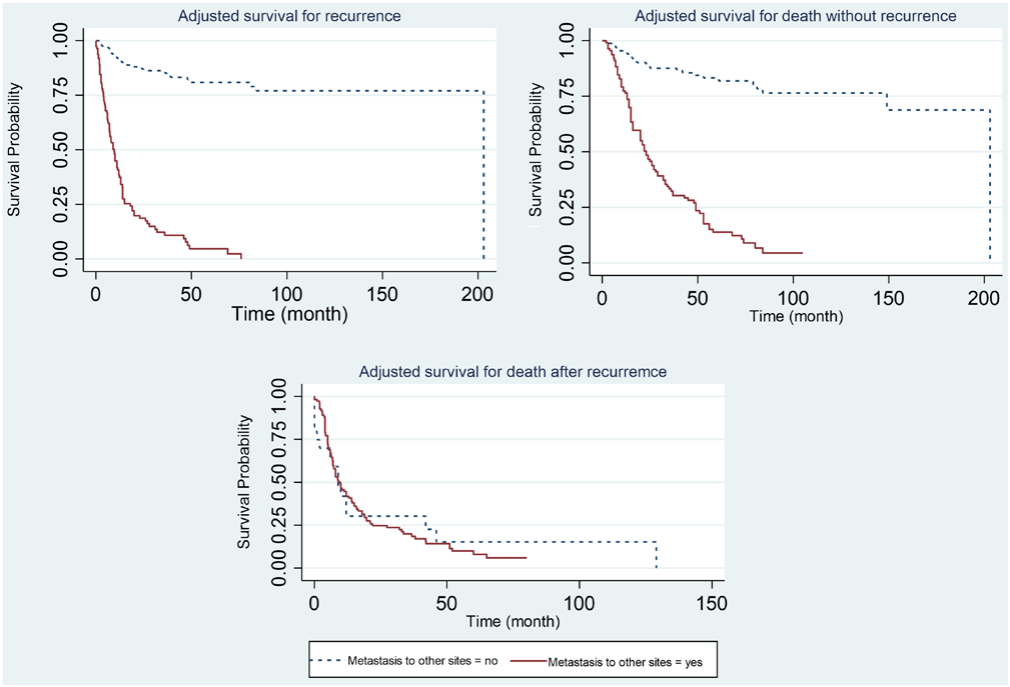
Adjusted survival probability for the events according to the metastasis to other sites.

**Figure 3.**
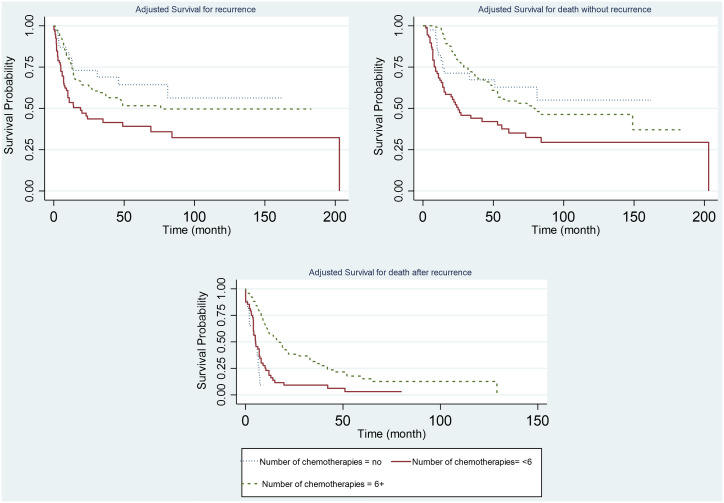
Adjusted survival probability for the events according to the number of chemotherapies.

**Figure 4.**
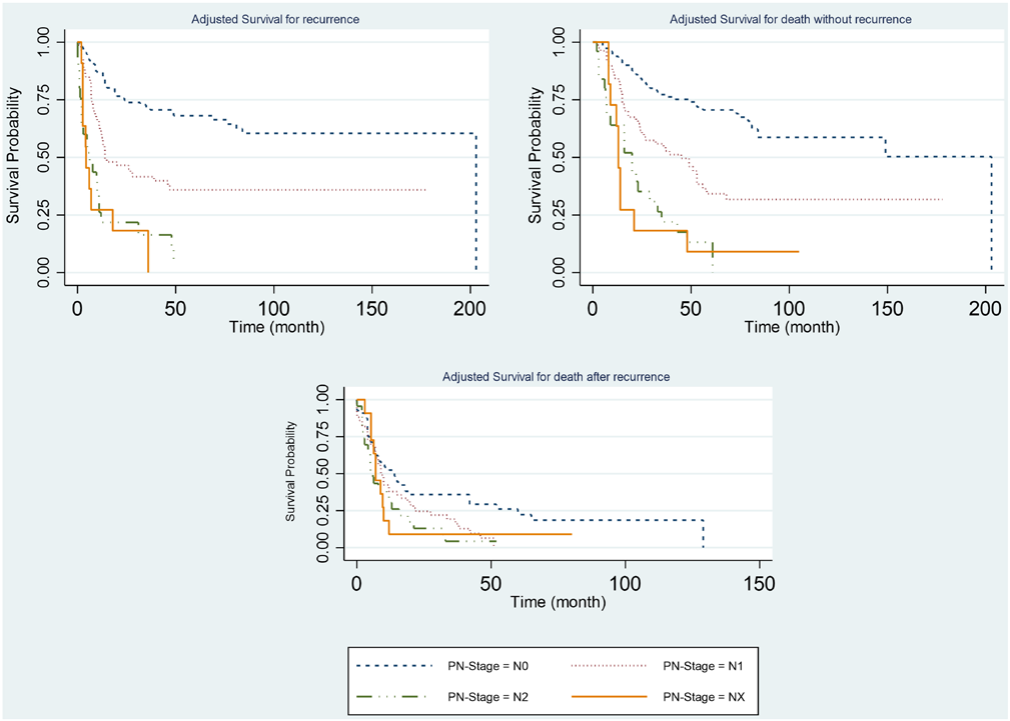
Adjusted survival probability for the events according to the pN stage.

Higher recurrence and death rates were observed among patients with metastasis, among patients who had less than 6 chemotherapies, and among patients with higher pN stage (Figures 2-4).

## DISCUSSION

This study aimed to model the effect of demographics and clinical characteristics on recurrence and postoperative death in patients with CRC, utilizing a semi-competing risk analysis. The study showed that metastasis to other sites, higher pN stage, and undergoing fewer chemotherapies were associated with a raised risk of recurrence and death. Higher pN stage was associated with significantly higher hazards for recurrence, death without recurrence, and death with recurrence, while metastasis to other sites was associated with significantly higher hazards of recurrence and death after recurrence.

Recurrence after surgery is one of the major problems affecting the long-term survival of CRC patients. Often occurring within the first 2 years after surgery, recurrence is associated with poor survival outcomes in the first 5 years.^25^ In the present study, about 80% of patients who experienced a recurrence of disease after surgery died by the end of the study, while in patients who did not experience a recurrence, the mortality rate was 10.5%. The recurrence rate in the first 5 years post-curative resection has been reported to be 25%-37%.^11,12^

In line with our results, other studies showed a direct link between age and recurrence.^15^ Some studies have also demonstrated a significant connection with age and local and distant recurrence rates and excess mortality,^26^ although this was not demonstrated in a study by Kawai et al.^27^ Therefore, diagnosis of the disease at an early age can lead to a complete cure and reduce the risk of recurrence.

Metastasis to other sites showed a significant association with both nonterminal and terminal events, as was evident in similar studies,^26^ emphasizing that diagnosis of the disease before metastasis occurs is crucial in improving outcomes.

Similar to our findings, complementary treatment has been shown to have a significant preventive effect on recurrence and mortality and chemotherapy to effectively reduce recurrence,^28^ suggesting that chemotherapy can decline the hazard of recurrence and death.

Although curative surgical resection is an effective treatment in patients with CRC, poorer diagnosis is associated with later stages of the disease, and about 30% of patients have a recurrence after surgery,^4,29^ which is compatible with the results of this study. In other studies, the stage of disease was significantly related to death,^26^ recurrence,^26^ and death after recurrence.^11^

In the present study, we did not separate recurrences into early and late events in order to fulfill the minimum required sample size for the semi-competing risk analysis. There was no statistically significant difference in the median survival between early (<2 years) and late recurrence (>2 years) (results not shown). In other studies, no significant difference was observed between early and late recurrence.^30^

We utilized a semi-competing risk approach to model the risk factors of recurrence and death. This framework provides us with unbiased estimates of probabilities of the outcomes. Since, in studies like the present study, there is a strong correlation between the terminal and nonterminal events (in the present study: *r* = 0.92, *P* < .001), simple utilization of a conventional survival model leads to an overestimation of the terminal event rates. In the classical approaches, the terminal event is considered as an independent censoring mechanism, which is not the case in reality and would lead to an overestimation of the effects of the risk factor on the outcomes.^20,23^ Utilizing semi-competing risk analysis, we considered the dependence between the 2 events to be part of the model specifications and therefore avoid the bias.

The largest limitation of the study was the width of the CIs in some analyses, especially in the multivariate analysis, and the CIs for the non-computable parameter estimates. The small sample size in the subgroups is the likely major contributor to this limitation. The Bayesian approach in estimating parameters and their credibility intervals may be the solution. In addition, the introduction of nonterminal- and terminal-specific prediction nomograms into the results could be useful for risk assessment.^31^

## CONCLUSION

Identifying the factors affecting the recurrence of disease after surgery and the duration of recurrence until the death of patients can provide more appropriate treatment and follow-up strategies for high-risk patients.

## Figures and Tables

**Table 1.** Demographic and Clinical Profile of Patients with CRC by Nonterminal and Terminal Events

	Recurrence (n = 131)		Death (n = 121)		Total (n = 284)	
	Frequency	Percent	Frequency	Percent	Frequency	Percent
Age at diagnosis (years)						
							<50	39	29.8	35	28.9	91	32.0
							51-70	72	55.0	65	53.7	158	55.6
>70	20	15.3	21	17.4	35	12.3
Gender						
							Female	56	42.7	50	41.3	134	47.2
Male	75	57.3	71	58.7	150	52.8
BMI						
							Normal	23	17.6	22	18.2	46	16.2
							Overweight	80	61.1	72	59.5	180	63.4
Obese	28	21.4	27	22.3	58	20.4
Metastasis to other sites						
							No	21	16.0	27	22.3	173	60.9
Yes	110	84.0	94	77.7	111	39.1
Cancer site						
							Colon	86	65.6	76	62.8	185	65.1
Rectum	45	34.4	45	37.2	99	34.9
Surgery						
							No	15	11.5	11	9.1	27	9.5
Yes	116	88.5	110	90.9	257	90.5
Radiotherapy						
							No	87	66.4	80	66.1	195	68.7
Yes	44	33.6	41	33.9	89	31.3
Chemotherapy						
							No	12	9.2	12	9.9	41	14.4
Yes	119	90.8	109	90.1	243	85.6
Number of chemotherapy						
							No	12	9.2	12	9.9	41	14.4
							<6	43	32.8	48	39.7	95	33.5
6+	76	58.0	61	50.4	148	52.1
Morphology						
							No adeno	2	1.5	2	1.7	3	1.1
Adeno	129	98.5	119	98.3	281	98.9
Grade (differentiation level)						
							Well	44	33.6	44	36.4	117	41.2
							Moderate	76	58.0	66	54.5	145	51.1
Poor	11	8.4	11	9.1	22	7.7
Tumor size						
							<4	29	22.1	25	20.7	72	25.4
							≥4 and <7	74	56.5	71	58.7	160	56.3
≥7	28	21.4	25	20.7	52	18.3
Disease stage						
							B	28	21.4	23	19.0	133	46.8
							C	37	28.2	41	33.9	84	29.6
D	66	50.4	57	47.1	67	23.6
pT stage						
							T2	7	5.3	7	5.8	41	14.4
							T3	92	70.2	85	70.2	202	71.1
							T4	23	17.6	21	17.4	32	11.3
TX	9	6.9	8	6.6	9	3.2
pN stage						
							N0	55	42.0	44	36.4	165	58.1
							N1	43	32.8	45	37.2	83	29.2
							N2	22	16.8	22	18.2	25	8.8
NX	11	8.4	10	8.3	11	3.9

BMI, body mass index; CRC, colorectal cancer.

**Table 2.** The Rate of Nonterminal and Terminal Events that Occurred in Patients with CRC

	Recurrence (n = 131)		*P* ^#^	Death (n = 121)		*P* ^#^
	Rate (per 1000)	95% CI		Rate (per 1000)	95% CI	
Age at diagnosis (years)						
							<50	11.21	8.19-15.35	**.001**	8.27	5.94-11.52	**<.001**
							51-70	12.17	9.66-15.33			8.99		7.05-11.46
>70	38.22	24.66-59.23		26.38	17.20-40.46								
Gender						
							Female	11.64	8.95-15.12	.335	8.66	6.56-11.42	.235
Male	14.70	11.72-18.43		10.95	8.68-13.82								
BMI category						
							Normal	15.75	10.47-23.71	.579	12.32	8.11-18.71	.479
							Overweight	12.24	9.83-15.24			9.15		7.26-11.53
Obese	14.57	10.06-21.10		10.38	7.12-15.13								
Metastasis to other sites						
							No	2.46	1.60-3.77	**<.001**	2.98	2.04-4.34	**<.001**
Yes	79.58	66.00-95.92		29.46	24.07-36.06								
Cancer site						
							Colon	12.97	10.50-16.03	.853	9.20	7.35-11.52	.363
Rectum	13.68	10.22-18.33		11.26	8.41-15.09								
Surgery						
							No	24.19	14.58-40.11	.105	12.10	6.70-21.85	.767
Yes	12.48	10.40-14.97		9.69	8.04-11.68								
Radiotherapy						
							No	12.05	9.77-14.87	.499	9.18	7.37-11.42	.344
Yes	16.30	12.13-21.90		11.58	8.53-15.73								
Chemotherapy						
							No	6.86	3.90-12.09	.057	6.66	3.78-11.72	.321
Yes	14.57	12.17-17.43		10.43	8.64-12.58								
Number of chemotherapies						
							No	6.86	3.90-12.09	**.041**	6.66	3.78-11.72	**<.001**
							<6	17.40	12.91-23.41			16.38		12.35-21.74
≥6	13.34	10.65-16.70		8.11	6.31-10.42								
Morphology						
							No adeno	18.92	4.73-75.66	.499	17.09	4.28-68.35	.437
Adeno	13.15	11.06-15.62		9.80	8.19-11.73								
Grade differentiation level						
							Well	10.52	7.83-14.14	.155	8.77	6.52-11.78	.357
							Moderate	15.75	12.58-19.72			10.63		8.35-13.53
Poor	12.10	6.70-21.84		10.71	5.93-19.34								
Tumor size						
							<4	12.12	8.42-17.44	.410	8.75	5.91-12.95	.494
							<7	13.20	10.51-16.58			10.00		7.92-12.61
≥7	14.60	10.08-21.15		10.88	7.35-16.11								
Disease stage						
							B	4.29	2.96-6.21	**<.001**	3.14	2.09-4.73	**<.001**
							C	12.78	9.26-17.63			12.00		8.83-16.29
D	134.90	105.35-170.68		37.44	28.88-48.54								
pT stage						
							T2	3.71	1.77-7.79	**<.001**	3.54	1.69-7.43	**<.001**
							T3	12.54	10.22-15.39			9.47		7.65-11.71
							T4	37.73	25.07-56.78			19.61		12.79-30.07
TX	103.99	54.11-199.85		34.63	17.32-69.25								
pN stage						
							N0	7.90	6.07-10.29	**<.001**	5.37	4.00-7.22	**<.001**
							N1	16.87	12.51-22.75			13.88		10.37-18.60
							N2	74.30	48.92-112.84			39.39		25.94-59.83
NX	99.50	55.10-179.67		37.73	20.30-70.13								

BMI, body mass index; CRC, colorectal cancer.

^#^ *P*-values computed from log-rank test and bold *P*-values indicate significant differences.

**Table 3.** Predictors of Nonterminal and Terminal Events Utilizing Univariable Semi-competing Risk Approach

		Recurrence		Death Without Recurrence		Death After Recurrence	
		HR	95% CI	HR	95% CI	HR	95% CI
Age at diagnosis (years)	Trend effect	2.064	1.324-3.216*	3.621	1.502-8.729*	1.376	0.869-2.18
Gender	Male	1.028	0.563-1.877	2.343	0.661-8.306	1.045	0.555-1.969
BMI category	Trend effect	1.200	0.731-1.970	2.068	0.759-5.636	0.837	0.514-1.362
Metastasis to other sites	Yes	36.260	20.018-65.680*	0.338	0.042-2.727	2.563	1.248-5.26*
Cancer site	Rectum	0.871	0.470-1.614	1.767	0.567-5.509	1.114	0.576-2.156
Surgery	Yes	0.429	0.172-1.074	0.572	0.062-5.271	1.404	0.511-3.859
Radiotherapy	Yes	0.915	0.487-1.720	1.217	0.376-3.94	0.773	0.394-1.517
Chemotherapy	Yes	2.358	0.932-5.960	1.201	0.267-5.397	0.326	0.117-0.908*
Number of chemotherapies	Trend effect	0.891	0.574-1.383	0.389	0.186-0.813*	0.296	0.177-0.494*
Morphology	Adeno	0.367	0.029-4.634	NC	NC	0.276	0.026-2.929
Grade (differentiation level)	Well	Referent	–	–	–	–	–
	Moderate	1.748	0.934-3.273	0.450	0.123- 1.637	1.618	0.816-3.21
	Poor	2.780	0.881-8.769	2.741	0.426-17.636	3.641	1.082-12.25*
Tumor size	Trend effect	1.510	0.954-2.390	1.678	0.694-4.053	1.234	0.764-1.995
Disease stage	B	Referent	–	–	–	–	–
	C	4.989	2.498-9.965*	4.204	1.33-13.289*	4.434	1.888-10.413*
	D	54.813	25.257-118.957*	3.054	0.30-31.036	6.505	2.678-15.803*
pT stage	T2	Referent	–	–	–	–	–
	T3	8.753	3.025-25.332*	3.108	0.666-14.508	1.800	0.843-3.841
	T4	25.120	6.993-90.244*	7.773	0.918-65.839	2.504	0.860-7.291
	TX	52.557	10.529-262.336*	NC	NC	5.005	1.483-16.886*
pN stage	N0	Referent	–	–	–	–	–
	N1	2.914	1.604-5.293*	4.033	1.259-12.926*	2.254	1.127-4.509*
	N2	14.859	5.849-37.747*	6.927	0.695-69.096	6.867	2.634-17.902*
	NX	11.062	3.652-33.506*	NC	NC	5.805	1.854-18.174*

Trend effect: The model considered the trend effect for ordinal categorical variables. The frailty components were significant in all univariable models (variance of frailties ranged between 0.195 and 2.84, and almost in all models, the 95% CI did not contain 1).

^*^ *P* < .05.

BMI, body mass index; HR, hazard ratio; NC, not computable.

**Table 4.** Predictors of Nonterminal and Terminal Events Utilizing Multivariable Semi-competing Risk Approach

		Recurrence		Death Without Recurrence		Death After Recurrence	
		HR	95% CI	HR	95% CI	HR	95% CI
Age at diagnosis (years)	Trend effect	1.309	0.926-1.852	2.354	0.970-5.711	0.805	0.564-1.148
Gender	Male	0.989	0.634-1.541	2.399	0.727-7.915	0.712	0.443-1.144
Metastasis to other sites	Yes	36.032	19.483-66.637*	0.105	0.010-1.122	2.669	1.241-5.744*
Number of chemotherapies	Trend effect	0.794	0.578-1.090	0.390	0.173-0.883*	0.317	0.205-0.491
Grade (differentiation level)	Well	Referent	–	–	–	–	–
	Moderate	1.539	0.992-2.390	0.318	0.093-1.083	1.559	0.936-2.597
	Poor	1.959	0.879-4.365	0.598	0.098-3.637	1.913	0.791-4.626
Tumor size	Trend effect	1.187	0.857-1.645	1.145	0.495-2.650	1.239	0.850-1.805
pT stage	T2	Referent	–	–	–	–	–
	T3	0.958	0.497-1.846	2.021	0.434-9.412	0.879	0.370-2.092
	T4	1.058	0.474-2.362	8.578	0.878-83.822	0.883	0.338-2.304
	TX	NC	NC	NC	NC	NC	NC
pN stage	N0	Referent	–	–	–	–	–
	N1	1.171	0.726-1.887	4.325	1.268-14.747*	1.593	0.930-2.730
	N2	2.455	1.323-4.555*	4.519	0.425-48.004	1.914	1.015-3.608*
	NX	NC	NC	NC	NC	NC	NC

The frailty component was significant in the multivariable model [variance of frailties: 0.179, 95% CI (0.051-0.63)]. Trend effect: The model considered the trend effect for ordinal categorical variables. The variables BMI category, cancer site, surgery, radiotherapy, chemotherapy, morphology, and disease stage could not be entered in the model in the multivariable model (all *P* > .05).

^*^ *P* < .05.

BMI, body mass index; HR, hazard ratio; NC, not computable.
